# Investigation into the protective effects of protocatechuic acid in bleomycin-induced pulmonary remodeling and fibrosis in rats: role of MMP-2/TIMP-1 and CTGF/NOX4 pathway

**DOI:** 10.1007/s00210-025-04410-6

**Published:** 2025-07-10

**Authors:** Rania Elgohary, Sahar Abd Elwahab, Abeer Salama

**Affiliations:** 1 Narcotics, Ergogenics and Poisons Department, Medical Research and Clinical Studies Institute, National Research Centre, (ID: 60014618), Dokki, Cairo Egypt; 2 Medical Biochemistry and Molecular Biology Department, Faculty of Medicine, Cairo University Al Kasr Al Aini, Old Cairo, Cairo Governorate, Egypt; 3 Pharmacology Department, Medical Research and Clinical Studies Institute, National Research Centre (ID: 60014618), Dokki, Cairo Egypt

**Keywords:** Pulmonary fibrosis, Bleomycin, ECM, Protocatechuic acid, TNF-α, CTGF/NOX4

## Abstract

Idiopathic pulmonary fibrosis (IPF) is an irreversible and progressive interstitial lung disease that results from excessive tissue repair. Production of excessive extracellular matrix (ECM) by myofibroblasts has been known as an important pathological feature in IPF. Connective tissue growth factor (CTGF) is a secreted matricellular protein modulating myofibroblast activation and ECM deposition, leading to fibrosis and tissue remodeling. Protocatechuic acid is extensively distributed in many edible nuts, vegetables, and fruits and is readily absorbed by both animals and humans. Numerous biological actions of protocatechuic acid (PCA) have been observed, including antibacterial, antidiabetic, antioxidant, and anti-inflammatory characteristics. The purpose of this study is to investigate the protective effect of PCA on bleomycin-induced pulmonary fibrosis in rats. The pathological changes of the lung and levels of malondialdehyde (MDA) and glutathione (GSH) were measured*.* Our results revealed that PCA decreased the oxidative production of lipid MDA and increased GSH content. Moreover, PCA suppressed the expression of inflammatory biomarkers transforming growth factor β (TGF-β) and tumor necrosis factor α (TNF-α), as well as decreased collagen deposition and ECM markers alpha-smooth muscle actin (α-SMA), matrix metalloproteinase-2 (MMP-2), and metallopeptidase inhibitor 1 (TIMP-1). PCA has an anti-fibrotic effect against pulmonary fibrosis by downregulation of the CTGF/NOX4/ET-1 gene expression. Also, PCA treatment ameliorated BLM-induced lung damage by improving alveolar sac structure, reducing inflammatory cell infiltration, and preserving bronchiolar epithelial integrity, suggesting that PCA may serve as a potential treatment option for PF.

## Introduction

Idiopathic pulmonary fibrosis (IPF) and many other diseases are associated with chronic inflammation. The inflammatory response is a self-contained and complex process that is accurately organized in order to prevent much serious damage to the body after numerous injuries. A damaging chronic inflammatory state results from the inappropriate organization of this defensive mechanism’s fundamental nature (Basu 2010). IPF is a diffuse parenchymal lung disease that affects between 2.8 and 9.3 instances out of every 100,000 people annually (Hutchinson et al. 2015). According to Noble et al., there are 20 new instances for every 100,000 individuals annually (Noble et al. 2012), and the median survival is typically only 3–5 years after diagnosis if no treatment is provided (Park et al. 2021).

IPF pathophysiology is influenced by fibroblast and myofibroblast accumulation and proliferation in the lung parenchyma as a result of recurrent alveolar epithelial micro-injuries (Raghu et al. 2011). Myofibroblasts are considered to be essential for the promotion of the fibrotic cycle and the perpetuation of injury because they play a crucial role in the deposition of ECM, the release of inflammatory mediators, and epithelial injury (Selman et al. 2001). Increased myofibroblast and other cell type deposition of ECM components like collagen and elastin causes uncontrollably large lung tissue scarring, which leads to lung function loss (Bormann et al. 2022). Matrix metalloproteinases (MMPs) are considered a family of zinc-dependent endopeptidases that play a key role in the breakdown of ECM. Tissue inhibitors of MMPs (TIMPs) are upregulated during tissue injury, lung development, and host defense (Parks and Shapiro 2001).

Continued cytokine transforming growth factor-β (TGF-β) activation plays an essential role in the myofibroblast phenotype maintaining (Border and Noble 1994). TGF-β is a crucial mediator of the development and progression of fibrosis. TGF-β upregulation has been shown in the experimental pulmonary fibrosis model mouse (Blobe et al. 2000). In normal physiology, TGF-β regulates multiple functions, including cell proliferation and differentiation, immunomodulation, and apoptosis (Aschner and Downey 2016). One important candidate in the pathophysiology of IPF is NADPH oxidase 4 (Nox4); it is overexpressed in the lungs of both humans and experimental animals after BLM treatment. The primary factor influencing NOX4 expression is TGF-β. NOX4 regulates the expression of collagen and α-SMA in lung fibroblasts (Fang et al. 2021). NADPH oxidases (NOXs) work as the primary source of ROS, catalyzing the O2 reduction. Specifically, NOX4 is closely associated with myofibroblast activation and the regulation of the lung fibroblast phenotype. Additionally, TGF-β triggers the expression of two essential matricellular proteins, connective tissue growth factor (CTGF), and endothelin-1 (ET-1), which function in tandem with this cytokine to promote fibrogenic reactions, including the creation of myofibroblasts (Mehdizadeh et al. 2022).

Bleomycin (BLM), a chemotherapeutic glycoprotein antibiotic, is used in therapeutic chemotherapy for the management of curable malignancies. Life-threatening and irreversible pulmonary fibrosis is linked to elevated cumulative doses of BLM and is also associated with a substantial morbidity and mortality rate of 1–3% (Froudarakis et al. 2013). Particularly, BLM-induced pulmonary fibrosis (PF) murine models demonstrated an increase in the expression of MMPs and TIMPs (Pardo et al. 2016a).

Notably, PF is a significant side effect of infection with the new coronavirus, severe acute respiratory syndrome coronavirus-2 (SARS-CoV-2), and worsens the quality of life for COVID-19 patients. Many risk variables, such as age and gender, are shared by both PF and virally induced acute respiratory distress syndrome (ARDS). Additionally, lung epithelial cells and fibroblasts were involved in COVID-19–induced PF (Wang et al. 2020). Therefore, a unique approach to treating severe COVID-19 and averting potential long-term fibrotic repercussions after the present pandemic is to look into the pathophysiology of PF and hunt for new therapeutic targets.

Protocatechuic acid (PCA) is classified as a dihydroxybenzoic acid and is a member of the phenolic acid structural family. PCA has numerous advantageous antibacterial, anti-inflammatory, antiviral, antioxidative, hepatoprotective, and cardioprotective properties and is found in fruits, vegetables, and grains (purple rice bran) (Choi and Kim 2022). However, the role and mechanism of PCA in BLM-induced PF remain still unknown. The goal of the current study was to assess PCA’s anti-fibrotic impact in treating PF in vivo and to highlight the potential underlying processes.

## Materials and methods

### Chemicals

Bleomycin hydrochloride was purchased from Sigma Aldrich Co., Saint Louis, Missouri, USA, and protocatechuic acid was purchased from Santa Cruz, CA, USA. All other chemicals were of the highest analytical grade available.

### Animals

Adult male albino 200–250 g Wistar rats were purchased from the National Research Center’s animal house in Giza, Egypt, and were provided with free-flowing tap water and a standard lab diet. The experimental animals were housed in a climate-controlled environment with a 12-h light/dark cycle and a temperature range of 22 to 25 °C. The National Institutes of Health’s Guide for the Care and Use of Laboratory Animals (NIH No. 85:23 revised 1985) is in accordance with the guidelines provided by the CPCSEA and the World Medical Association Declaration of Helsinki on Ethical Principles for studies involving experimental animals. All experimental procedures were conducted in compliance with the Animal Research: Reporting of In Vivo Experiments (ARRIVE) guidelines.

### Experimental design

Following a week of acclimatization, male albino Wistar rats were divided into four groups, each with ten rats, at random.

- **Group 1:** Rats received saline intranasally for 7 days and p.o daily for 21 days and served as negative control.
- **Group 2**: Rats received 0.3 mg/kg of BLM intranasally for 7 days and served as fibrotic control (Raras et al. 2021).
- **Group 3:** Rats received BLM + PCA (25 mg/kg, orally) daily for 21 days (Salama et al. 2023).
- **Group 4:** Rats received BLM + PCA (50 mg/kg, orally) daily for 21 days.

Bleomycin hydrochloride 0.3 mg/kg was administered intranasally on days 1, 3, 5, 7, 9, 10, and 13 at a dose of 50 µL per nostril. Rats with successful persistent pulmonary fibrosis have previously been created using an administration strategy (Guzel-Seydim et al. 2011). On day 21, all rats were sacrificed via cervical dislocation. An ice-cold phosphate buffer (pH 7.4) was added to the dissected lung after it had been cleaned with saline. A tissue homogenizer (MPW-120, BitLab Medical Equipment, Poland) was used to generate a 20% homogenate. After that, homogenized tissues were centrifuged using a cooling centrifuge (Laboratory Centrifuge, 2 K15, Sigma Co., Germany) at 3000 rpm/min for 15 min at 4 °C. The supernatant was collected, stored at − 80 °C, and used in biochemical analysis (Salama et al. 2022).

### Biochemical analysis

Using commercially available kits, the MDA and GSH levels in the lung homogenate were determined (Biodiagnostic, Egypt) (Salama and Elgohary 2021). Collagen I (COL I), alpha-smooth muscle actin (α-SMA), metalloproteinase-2 (MMP2), tissue inhibitors of metalloproteinase-1 (TIMP-1), tumor necrosis factor-alpha (TNF-α), and transforming growth factor (TGF-β) levels were measured using ELISA kits acquired from Sunlong Biotech Co., Ltd., China.

### Quantitative real‑time PCR gene expression of NOX4, CTGF, and ET-1 in rat’s lung tissues

According to Chomczynski and Sacchi, total RNA was extracted from rats’ lung tissue homogenates using TRI reagent (Molecular Research Center Inc., Cincinnati, OH) (Chomczynski and Sacchi 1987). Quantification of the extracted RNA was done by spectro-photometry (JENWAY, USA) at 260 nm. The obtained RNA is utilized for assessment of NOX4, CTGF, and ET-1 gene expression levels with quantitative RT-PCR in agreement with the manufacturer’s protocol (GoTaq® 1-Step RT-qPCR System). Cycling conditions were adjusted according to the manufacturer. PCR primers were designed with Gene Runner Software (Hastings Software Inc., Hastings, NY, USA) with RNA sequences obtained from GenBank, using GAPDH as a housekeeping gene (Table 1). The obtained data were calculated using the 2^−ΔΔCT^ method (Livak and Schmittgen 2001).

**Table 1:** Primer sequences used for RT-qPCR

Gene	Forward primer	Reverse primer
NOX4	5′-AGTCAAACAGATGGGA-3′	5′-TGTCCCATATGAGTTGTT-3′
CTGF	5′-TGTGAAGACATACAGGGCTAA-3′	5′-GTTCTCACTTTGGTGGGATAG-3′
ET-1	5′-GAACATCTGTCCGGCTTCTAC-3′	5′-TATGGAATCTCCTGGCTCTCT-3′
GAPDH	5′-TGAACGGGAAGCTCACTGG-3′	5′-TCCACCACCCTGTTGCTGTA-3′

### Statistical analysis

All the values are presented as means ± SD. Data of this study were evaluated by one-way analysis of variance followed by Tukey’s multiple comparisons test. GraphPad Prism software, version 5 (Inc., San Diego, USA) was used to carry out these statistical tests. The difference was considered significant when *p* < 0.05.

### Histological examination

Different groups’ dissected lungs were preserved in 20% formalin. After 1 or 2 days of fixation, the tissue was dehydrated in increasing concentrations of alcohol (70%, 90%, and three changes in absolute alcohol), cleared with xylene, impregnated three times with soft paraffin at 50 °C, and then embedded in paraffin wax to create solid blocks containing the tissue. Seven millimeter-thick serial transverse sections were cut. Hematoxylin and eosin was used to stain the paraffin slices that were mounted on glass slides and coated in albumin glycerin. Under light microscopy, hematoxylin and eosin sections were examined qualitatively. To evaluate inflammatory cell infiltration and smooth muscle layer thickness, sections were stained with hematoxylin and eosin. Inflammatory cell infiltration was scored using the following scale: 0 = no inflammatory cell infiltration, 1 = inflammatory cells present centrally, 2 = infiltration centrally and some spread to the parenchyma, and 3 = massive infiltration (Sjöberg et al. 2017).

## Results

### Effects of protocatechuic acid on GSH and MDA lung contents in bleomycin-induced pulmonary fibrosis in rats

**Fig. 1:**
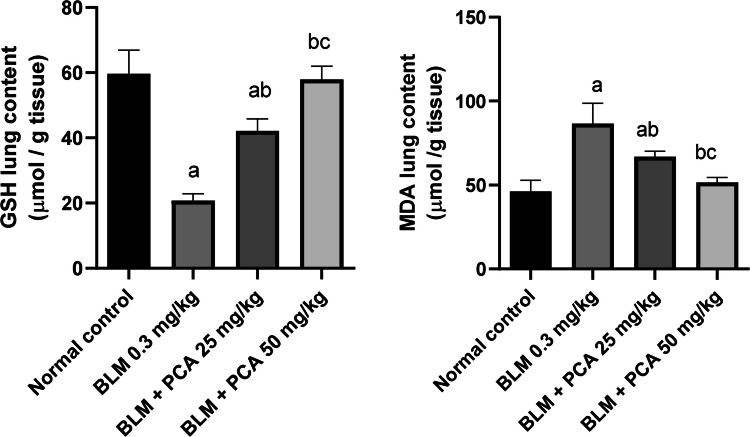
Effects of protocatechuic acid on GSH and MDA lung contents in bleomycin-induced pulmonary fibrosis in rats. Data were expressed as mean ± SD. Statistical analysis was carried out by one-way ANOVA followed by Tukey HSD test for multiple comparisons. ^a^Significantly different from normal control. ^b^Significantly different from BLM control..^c^Significantly different from BLM + PCA acid 25 mg/kg at *P* < 0.05

MDA concentration was increased by 88% in BLM-treated rats as compared to normal control. Both doses of protocatechuic acid caused a decline in MDA level by 23% and 41%, respectively, when compared to the BLM- challenged group (*P*-value < 0.05). GSH was also affected by BLM; its activity was decreased by 65% as compared to control (*P*-value < 0.05). Treatment with protocatechuic acid increased the activity of GSH by 103% and 180% compared to BLM control (*P*-value < 0.05) (Fig. 1).

### Effects of protocatechuic acid on TGF-β and TNF-α lung contents in bleomycin-induced pulmonary fibrosis in rats

**Fig. 2:**
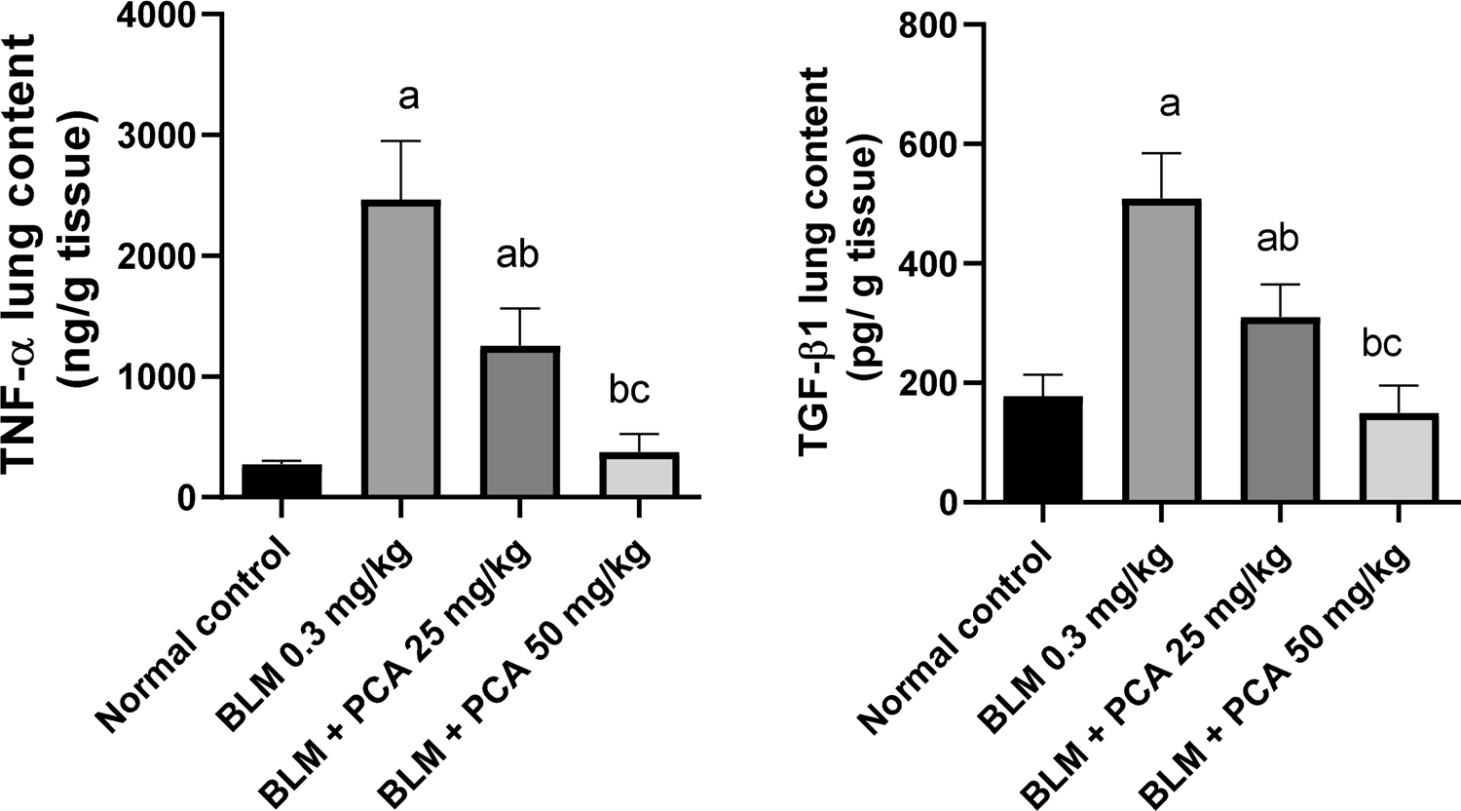
Effects of protocatechuic acid on TGF-β and TNF-α lung contents in bleomycin-induced pulmonary fibrosis in rats. Data were expressed as mean ± SD. Statistical analysis was carried out by one-way ANOVA followed by Tukey HSD test for multiple comparisons. ^a^Significantly different from normal control. ^b^Significantly different from BLM control. ^c^Significantly different from BLM + PCA acid 25 mg/kg at *P* < 0.05

BLM injection elevated TGF-β and TNF-α lung contents by 187% and 801% as compared to normal control (*P*-value < 0.05). Both doses of protocatechuic acid reduced their lung content by 39% and 71%, 49% and 85%, respectively, compared to the BLM- challenged group (*P*-value < 0.05) (Fig. 2).

### Effects of protocatechuic acid on collagen-1, α-SMA, MMP-2, and TIMP-1 lung contents in bleomycin-induced pulmonary fibrosis in rats

**Fig. 3:**
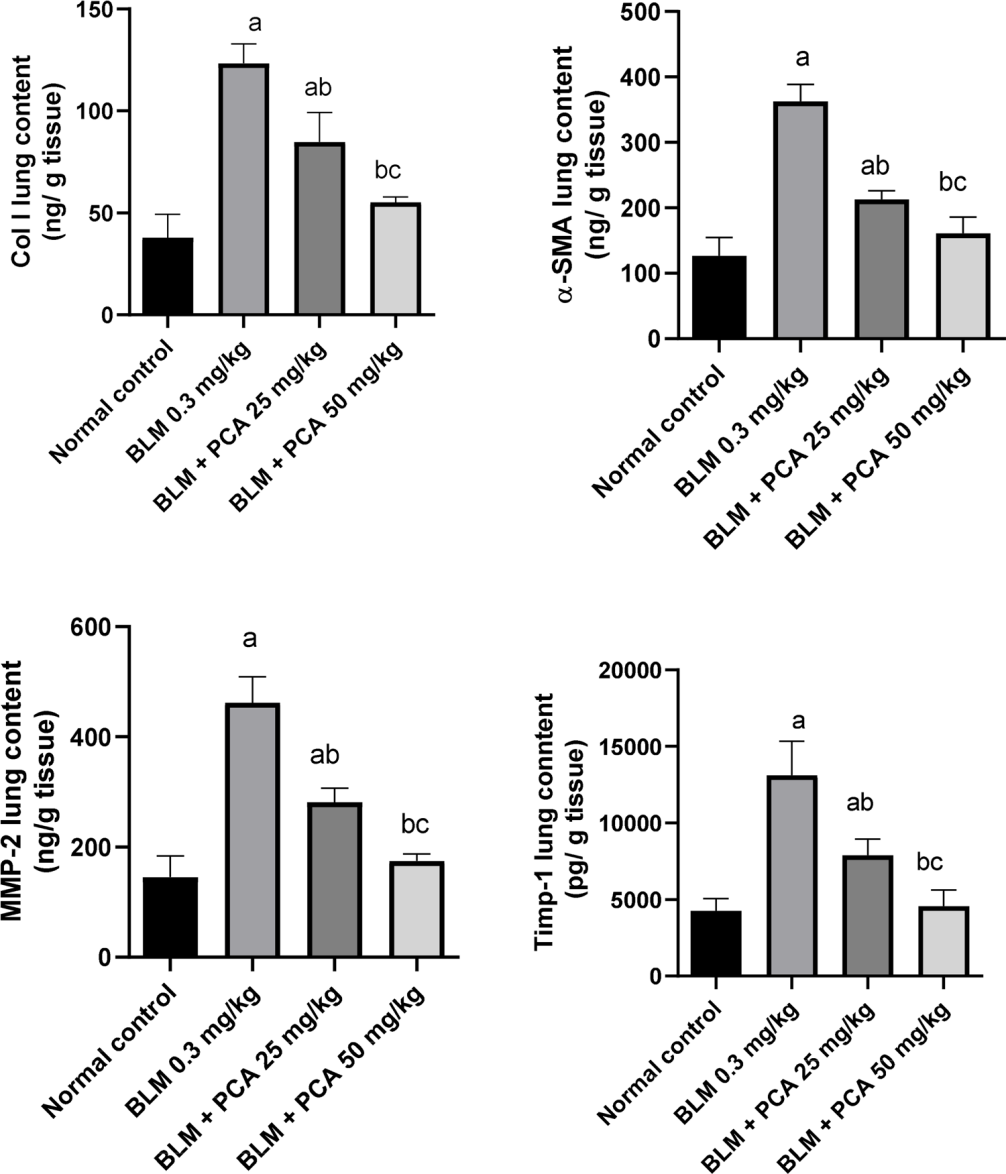
Effects of protocatechuic acid on collagen-1, α-SMA, MMP-2, and TIMP-1 lung contents in bleomycin-induced pulmonary fibrosis in rats. Data were expressed as mean ± SD. Statistical analysis was carried out by one-way ANOVA followed by Tukey HSD test for multiple comparisons. ^a^Significantly different from normal control. ^b^Significantly different from BLM control. ^c^Significantly different from BLM + PCA acid 25 mg/kg at *P* < 0.05

BLM injection elevated collagen-1 and α-SMA lung contents by 227% and 187% as compared to normal control (*P*-value < 0.05). Both doses of protocatechuic acid reduced their lung content by 31% and 55%, 41% and 56%, respectively, compared to the BLM-challenged group (*P*-value < 0.05) (Fig. 3).

MMP-2 and TIMP-1 lung contents were elevated in BLM-treated rats by 218 and 207% as compared to normal control (*P*-value < 0.05). Both doses of protocatechuic acid exhibited a reduction in lung contents of MMP-2 by 39% and 62% and TIMP-1 by 40% and 65%, respectively, compared to the BLM-challenged group (*P*-value < 0.05) (Fig. 3).

### Effects of protocatechuic acid on NOX-4, CTGF, and ET-1 gene expression in bleomycin-induced pulmonary fibrosis in rats

**Fig. 4:**
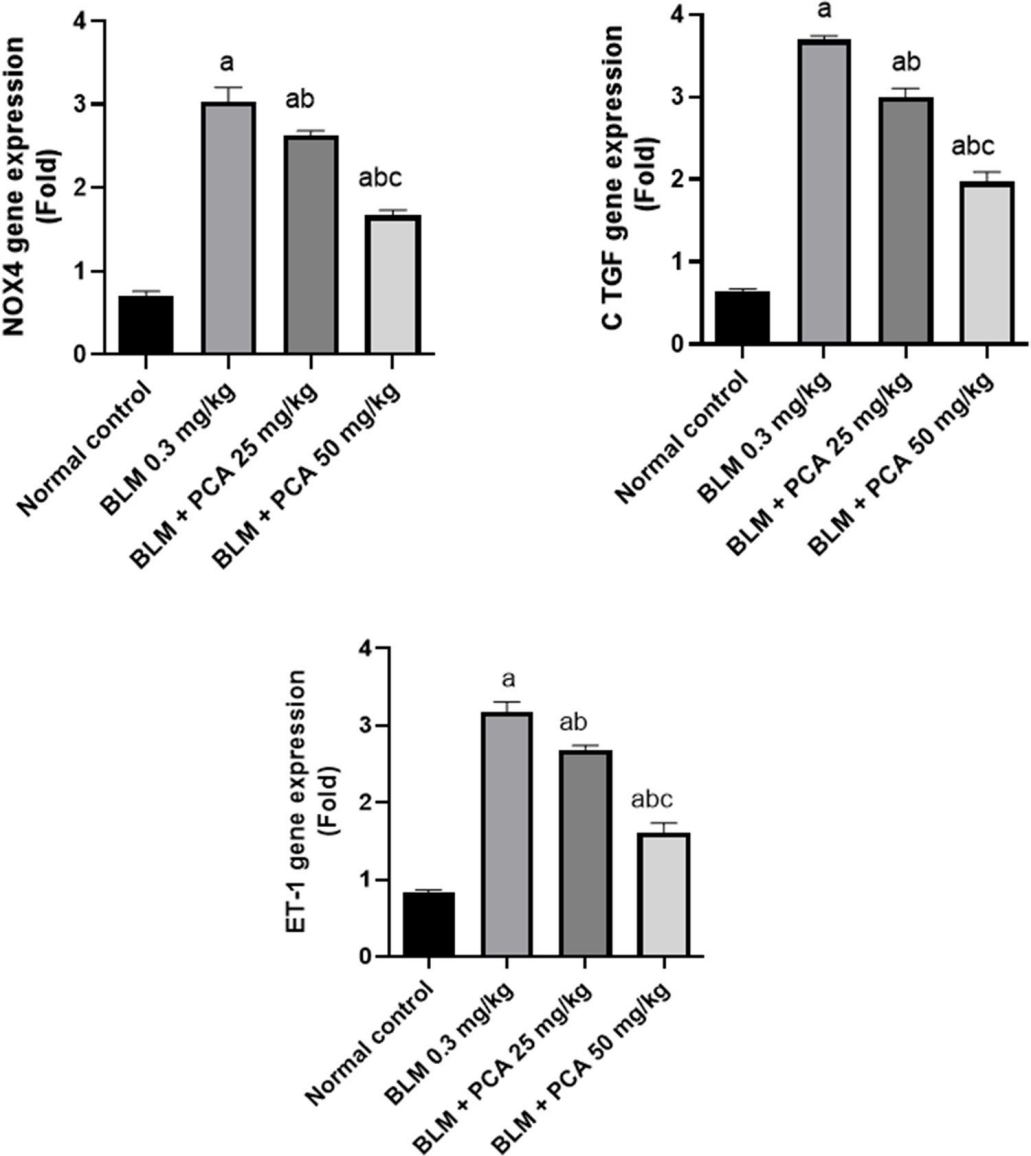
Effects of protocatechuic acid on NOX-4, CTGF, and ET-1 gene expression in bleomycin-induced pulmonary fibrosis in rats. Data were expressed as mean ± SD. Statistical analysis was carried out by one-way ANOVA followed by Tukey HSD test for multiple comparisons. ^a^Significantly different from normal control. ^b^From normal control. ^b^Significantly different from BLM control. ^c^Significantly different from BLM + PCA acid 25 mg/kg at *P* < 0.05

Administration of BLM showed a significant increase in the NOX-4, CTGF, and ET-1, as compared to the control group. While, protocatechuic acid 25 and 50 mg/kg administrations resulted in a significant decrease in the three parameters as compared to BLM control group (Fig. 4).

### Histopathological results

**Fig. 5:**
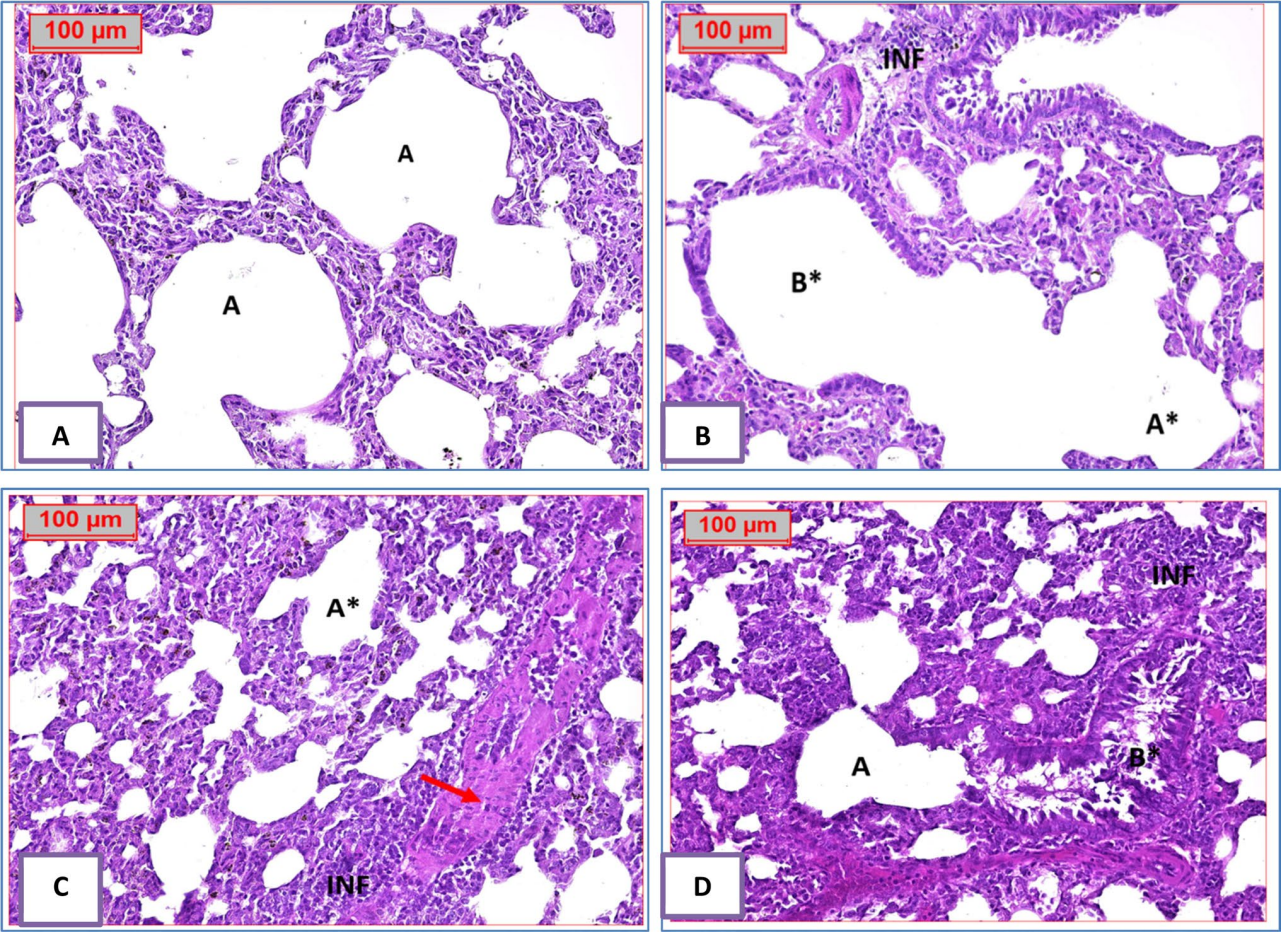
Effect of treatment with protocatechuic acid on lung histopathology in bleomycin-induced pulmonary fibrosis in rats. **A** A photomicrography of lung tissue **(G1: normal group)** showing normal-looking architecture, the alveolar sacs **(A)** will be separated by alveolar septa, comprising alveolar lining cells and thin-walled capillaries. The bronchiole **(B)** has intact wall with normal-looking ciliated columnar epithelium. **B** A photomicrography of lung tissue **(G2: BLM group)** showing the alveolar sacs totally destructed (A*). The bronchiole (B*) has damaged wall by inflammatory cells infiltrate **(INF)**. **C** A photomicrography of lung tissue **(G3: BLM + PCA 25 group)** showing destructed alveolar sacs (A*); some of them are separated by alveolar septa, infiltrated by inflammatory cells **(INF)**, and normal-looking bronchial vessels **(red arrows)**. **D** A photomicrography of lung tissue **(G4: BLM + PCA 50 group)** showing improvement in the alveolar sacs (A); separated by alveolar septa, some of them infiltrated by inflammatory cells (**INF)**. The bronchiole (B*) has intact wall with normal-looking ciliated columnar epithelium, with intra-bronchial inflammatory cells infiltrate with prominent macrophages **(H&E 200x)**

**Fig. 6:**
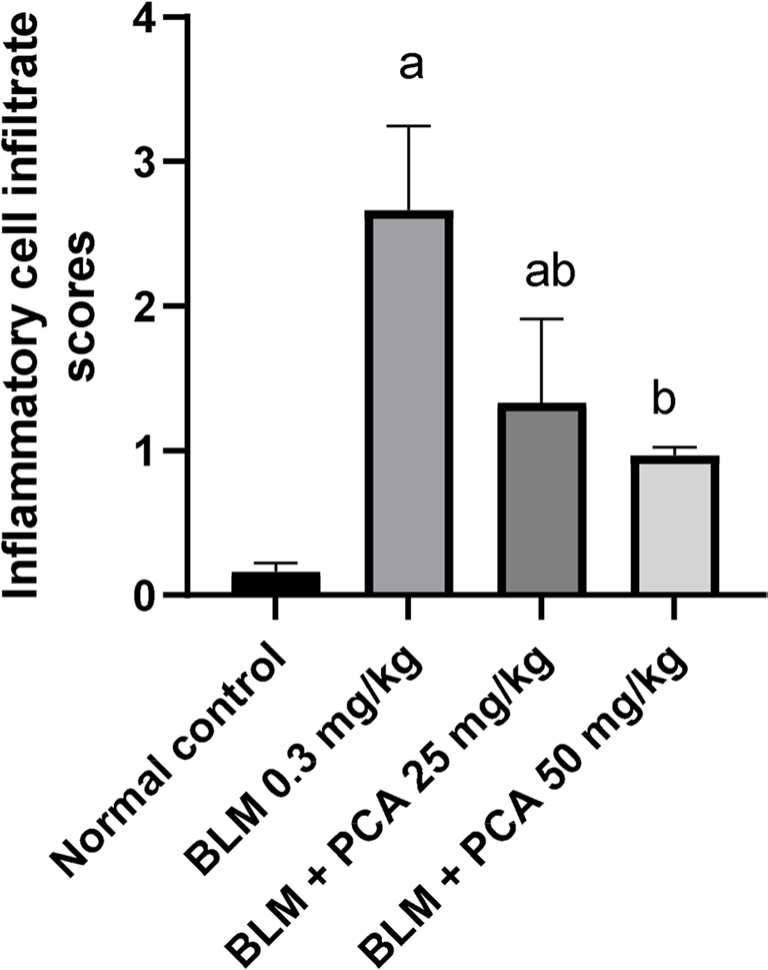
Quantification of inflammatory lung cell infiltrate. Data were expressed as mean ± SD. Statistical analysis was carried out by one-way ANOVA followed by Tukey HSD test for multiple comparisons. ^a^Significantly different from normal control. ^b^From normal control. ^b^Significantly different from BLM control. ^c^Significantly different from BLM + PCA acid 25 mg/kg at *P* < 0.05

A section of lung tissue **(G1: Control Normal Group)** showing normal architecture; the alveolar sacs are separated by alveolar septa, comprising alveolar lining cells and thin-walled capillaries. The bronchiole has an intact wall with normal ciliated columnar epithelium. A section of lung tissue **(G2: BLM)** showing the alveolar sacs totally destructed. The bronchiole has a damaged wall by inflammatory cells infiltrate. A section of lung tissue **(G3: BLM + protocatechuic acid 25)** showing destructed alveolar sacs; some of them are separated by alveolar septa, infiltrated by inflammatory cells, and normal-looking bronchial vessels. A section of lung tissue **(G4: BLM + protocatechuic acid 50)** showing improvement in the alveolar sacs **(A)**; separated by alveolar septa, some of them infiltrated by inflammatory cells. The bronchiole has an intact wall with normal-looking ciliated columnar epithelium, with intra-bronchial inflammatory cells infiltrated with prominent macrophages (Fig. 5). Inflammatory cell infiltrate scoring results are presented in Fig. 6.

## Discussion

Pulmonary fibrosis (PF) is an incurable and progressive lung disease showing scar tissue (fibrosis) to building up in the lungs, which makes it difficult to transport oxygen into the bloodstream effectively (Allen et al. 2020). Lung damage from IPF is irreversible, but medications can help slow down the progression of it and improve lung function (Spagnolo et al. 2021). The BLM-induced lung fibrosis is now the best model utilized to research putative pathways implicated in the etiology of pulmonary fibrosis and to develop treatment options, despite certain limitations with regard to recapitulation of human illness (Kolb et al. 2020). The present study divulged, for the first time, the effect of PCA on alleviating the pathological features of this disease by antioxidant, anti-inflammatory, and anti-fibrotic effects on BLM-induced pulmonary fibrosis in rats. The BLM model of lung fibrosis is characterized as the cheapest, easiest, fastest, most reproducible, and most extensively used animal model of PF. Moreover, it yields a fibrotic histologic picture similar to that of the human disease (Tashiro et al. 2017).

Several lines of evidence point to the critical role that oxidative stress plays in the pathological development of pulmonary fibrosis, and it has been documented that the pathophysiology of fibrosis is, at least in part, influenced by the generation of ROS. In clinical settings, BLM is a significant chemotherapeutic glycopeptide antibiotic used to treat various carcinomas and lymphomas (Aytemur et al. 2012). Even at therapeutic levels, PF is the most commonly reported side effect of BLM in cancer patients (Shariati et al. 2019). Since the lung lacks BLM hydrolase, which breaks down the carboxy amide link of the β-amino alanine moiety of BLM, the toxicity of BLM is seen in the lung (Zohny et al. 2022). BLM increases the generation of reactive oxygen species (ROS), which damages DNA and damages epithelial cells. As a result of increased collagen production and the deposition of other matrix proteins brought on by ongoing lung exposure to BLM, the pulmonary function deteriorates significantly (Zaafan et al. 2019). According to earlier research, redox cycling of an iron-BLM complex is linked to BLM-induced lung toxicity. This complex then catalyzes the production of ROS and the eventual advancement of lipid peroxidation concurrent with a reduction in GSH (Bahri et al. 2017). In line with Bakirhan et al., it was reported that BLM administration was shown to cause oxidative stress by decreasing SOD and GPx activity while increasing MDA levels in lung tissues (Bakirhan and Parlar 2023). Reduction of oxidative stress should lead to less damage to the lungs. According to our research, BLM-induced lung damage significantly improved when PCA was administered. Moreover, PCA administration led to a large rise in GSH content and a considerable decrease in lung MDA, suggesting that PCA is helpful in preserving the oxidant-antioxidant equilibrium. Prior studies discovered that PCA suppressed lipid peroxidation in a variety of settings, including kidney injury after doxorubicin therapy and a mouse model of diabetes (Adedara et al. 2019; Molehin et al. 2019). By reducing free radicals, triggering the Nrf-2 pathway, and boosting GSH and its dependent enzyme activities, PCA demonstrates its antioxidant effect (Cheng et al. 2019). Moreover, ROS trigger a number of redox-sensitive signaling cascades, including nuclear factor-κB (NF-κB), activator protein-1 (AP-1), and mitogen-activated protein kinase (MAPK). Growth factors, profibrotic cytokines, and inflammatory cytokines are all upregulated when these factors are activated. According to Cui et al. (2019), this is one of the main reasons why lung injuries occur (Cui et al. 2019).

Hence, one of the main signs of PF is persistent inflammation, which is accompanied by a rise in the quantity of inflammatory cells in the interstitial tissue and alveolar space. ROS and other cytokines are produced by activated inflammatory cells, and these molecules cause fibroblast and myofibroblast migration and proliferation (Checa and Aran 2020). Damaging alveolar epithelial cells in the early stages of the BLM-induced pulmonary fibrosis model in rats resulted in a significant release of pro-inflammatory fibrosis biomarkers and enhanced neutrophil infiltration, which exacerbated the inflammatory response in the lungs. Later in the course of the illness, there will be persistent inflammation dominated by lymphocyte infiltration (Liu et al. 2017). Thus, it is beneficial to suppress the inflammatory response in order to prevent pulmonary fibrosis from worsening. The pro-fibrotic and inflammatory mediator TNF-α causes damage to lung tissue and is crucial in the development and course of pulmonary fibrosis (Lu et al. 2019). Therefore, we measured the levels of this cytokine in the lung supernatant. Herein, the experimental results showed a significant elevation in TNF-α level after BLM stimulation. On the other hand, PCA significantly and dose-dependently reduced its level. Consistent with our results, a previous study showed that PCA treatment significantly downregulated the inflammatory markers such as NF-κB and TNF-α in the pulmonary damage induced by cyclophosphamide in rats (Salama et al. 2023).

In the initial phases of PF, macrophages play a pivotal role in this process by generating cytokines like TGF-β, establishing a fibrotic microenvironment, and stimulating the build-up of ECM, fibroblast differentiation into myofibroblasts, and fibroblast autophagy suppression (Mei et al. 2021). One of the main events in the pathophysiology of PF is the differentiation of lung fibroblasts into myofibroblasts. These cells are characterized by the expression of α-SMA and increased production and secretion of ECM proteins, such as collagen I and collagen III, which subsequently exacerbate the development of PF (Frangogiannis 2020). Animal model studies demonstrate that the expression of TGF-β1 is elevated in PF caused by BLM (Wynn 2011). In this study, we demonstrate that PCA showed a significant decrease in the levels of TGF-β1, Col I, and α-SMA. A recent study supported our results, showing that protocatechuic acid–mediated suppression of cardiac fibrosis was ameliorated by TGF-β1, which induced upregulation of Col1 in vivo or cardiac fibroblasts in neonatal rats (Song and Ren 2019). Our findings suggest that the protective effect of PCA against PF may be regulated through the TGF-β1 signaling pathway, suggesting that PCA treatment significantly preserved the equilibrium between the local lung microenvironment’s ECM component degradation, which is crucial for PF amelioration.

TGF-β1 and oxidative stress signals are significantly correlated throughout the fibrosis process, and TGF-β1 raises the levels of ROS in fibrotic pulmonary tissue (Cheresh et al. 2013). The key process in cell membranes and ROS products is the conversion of NOX to oxygen. There are seven NOX homologs in the NOX family. Of the seven NOX family members, NOX4 is most frequently linked to a range of fibrotic disorders (Veith et al. 2019). NOX4 functions as a mediator downstream of TGF-β1-mediated profibrotic reactions (Crestani et al. 2011). Moreover, CTGF is another candidate that is implicated in fibrotic diseases and activated by TGF-β1 in an ROS-dependent manner. CTGF is widely recognized as a downstream effector of TGF-β1 and functions as a key mediator that bridges TGF-β1 signaling and ECM protein accumulation. Upon TGF-β1 stimulation, CTGF is rapidly upregulated as an early response gene, contributing to fibroblast proliferation, ECM deposition, and tissue remodeling. Therefore, CTGF serves not only as a functional intermediate but also amplifies TGF-β1-driven fibrogenic responses (Yin et al. 2024). Also, Nox4 aids in the production of basal cellular ROS to sustain cellular processes like differentiation, proliferation, and oxygen sensing. However, dysregulated Nox4 expression is essential for the production of CTGF and illnesses associated with fibrosis (Bäck and Hansson 2015). Considering the important role of NOX4-mediated oxidative stress in pulmonary fibrosis, in our study, we tried to explain PCA antifibrotic effects from the viewpoint of NOX4 function and oxidative stress process. Administration of PCA significantly lowered BLM-induced levels of NOX4 and CTGF in the lungs, showing strong antioxidative effect. In vitro, by inhibiting the NOX4/ROS/p38 signaling pathway, PCA prevents the angiotensin II-induced proliferation and migration of cardiac fibroblasts (Song and Ren 2019). A previous study supports these findings (Chen et al. 2019), which revealed that in mesenchymal stem cells generated from bone marrow, PCA exhibits a dose-dependent antioxidant function via the production of free radical adducts, H +, and electron transfer.

The fact that ET-1 is a strong inducer of fibroblast chemotaxis, proliferation, and collagen synthesis is well known. Numerous cell types found in the lung, such as fibroblasts, alveolar macrophages, endothelium, and epithelial cells, are known to generate ET-1 (Mutsaers et al. 1998). Furthermore, in response to damage, endothelial cells have been stimulated to release more ET-1 (O'Brien et al. 1987) and stimulation with mediators including TGF-β1, TNF-α, and interleukin (IL)−1β, which are present in high amounts in the lungs of patients with PF (Southcott et al. 1995). The most likely cause of the elevated ET-1 levels is an increase in its synthesis by both resident lung and inflammatory cells that enter the lung after BLM injection. This is in line with human research on PF, which has shown elevated levels of ET-1 in the lungs of systemic sclerosis (SSc) patients (Cambrey et al. 1994). After BLM injection, the lung tissue of mice shows increased levels of ET-1, which indicates damage and problems with the endothelial cells (Zhao et al. 2023). We found that ET-1 expression significantly downregulates not only TGF-β1 and its downstream effectors but also other important fibrogenic factors, highlighting the multifactorial molecular mechanisms involved in the antifibrotic effects of PCA in an experimental model of PF.

**Fig. 7:**
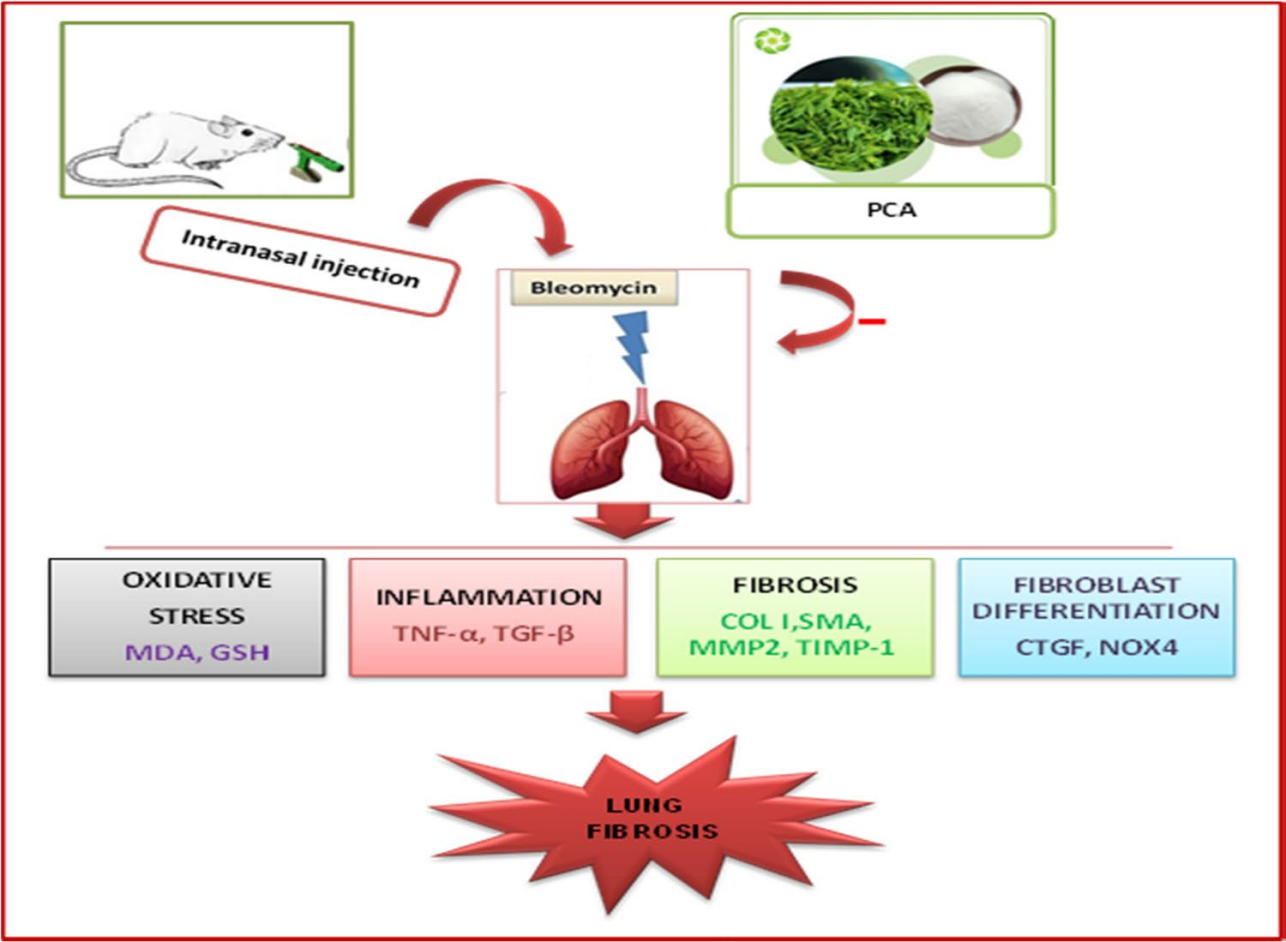
A summary of the protective effects of protocatechuic acid in bleomycin-induced pulmonary remodeling and fibrosis in rats. Role of MMP-2/TIMP-1 and CTGF/NOX4 pathway. Protocatechuic (PCA), malondialdehyde (MDA), glutathione (GSH), collagen I (COL I), alpha-smooth muscle actin (α-SMA), metalloproteinase-2 (MMP2), tissue inhibitors of metalloproteinase-1 (TIMP-1), tumor necrosis factor-alpha (TNF-α), transforming growth factor (TGF-β), NADPH oxidase 4 (NOX4), connective tissue growth factor (CTGF), and endothelin-1 (ET-1)

The majority of MMPs and TIMPs are thought to be implicated in the pathophysiological mechanisms of PF. Specifically, mice with genetic deletions in certain MMPs had less lung fibrosis following BLM administration compared to wild-type mice, while murine models of BLM-induced pulmonary fibrosis showed enhanced expression of MMPs and TIMPs (Pardo et al. 2016b). MMPs can also specifically cleave ECM domains, which are crucial in controlling extracellular inflammatory cytokines and chemokines. This allows MMP-9 to release forms that are bound to the surface and modulate immunological responses, including TNF-α (Gearing et al. 1995), or through degradation, such as IL-1β by MMP-2 (Gill et al. 2010). Prior research has demonstrated that TIMP-1 and MMPs play major roles in the remodeling and degradation of ECM. MMPs encourage the growth of PF, whereas TIMP-1 may control its expression (Churg et al. 2012). In line with the findings of Cinetto et al., it showed that the bronchoalveolar lavage fluid (BALF) of mice treated with BLM had higher levels of MMP-2, TIMP-1, MMP-9, and TIMP-2 (Cinetto et al. 2021). Hence, the inhibitory effect of PCA on MMPs and TIMP-1 has been investigated in the present study. This finding was compatible with a previous study that reported that PCA prevented cardiomyocyte loss and fibrosis by decreasing TGF-β1 and MMP-9 in myocardial infarction mediated by β-adrenergic agonist (Li et al. 2023). Thus, protocatechuic acid exhibits a promising anti-fibrotic effect against pulmonary fibrosis by attenuating oxidative stress, reducing inflammation, and suppressing fibrotic markers and gene expression related to fibrosis. These findings highlight its potential as a therapeutic candidate for managing pulmonary fibrosis (Fig. 7).

Finally, histopathological evaluation of lung tissue provided evidence for the ameliorative effect of PCA on BLM-induced pulmonary toxicity in rats. PCA protected lung tissue and reduced the damaging effects of BLM, showing improvement in the alveolar sacs and reduction of the inflammatory cells. Moreover, the bronchiole has an intact wall with a normal-looking ciliated columnar epithelium.

## Conclusion

Taken together, the results of the current study demonstrated for the first time that PCA can ameliorate BLM-induced pulmonary fibrosis by inhibiting the early inflammatory response, decreasing TGF-β1 and TNF-α contents. Moreover, PCA caused amelioration of ECM deposition via the inhibition of the MMP-2/TIMP-1/Col I/α-SMA/NOX4 and CTGF signal pathway. To conclude, our study indicates a therapeutic potential of PCA in the treatment of PF, and this could warrant further investigation.

## Data Availability

All source data for this work (or generated in this study) are available upon reasonable request.
